# The development of hyperbaric oxygen therapy for skin rejuvenation and treatment of photoaging

**DOI:** 10.1186/2045-9912-4-7

**Published:** 2014-04-01

**Authors:** Bralipisut Asadamongkol, John H Zhang

**Affiliations:** 1 Department of Physiology, Loma Linda University School of Medicine, Loma Linda, CA 92350, USA

**Keywords:** Hyperbaric oxygen therapy, Wrinkles, Mechanisms, Photoaging, Angiogenesis, UVB

## Abstract

Hyperbaric oxygen therapy (HBOT), a therapy that have patients breath in pure oxygen in a pressurized chamber, has been long used as a treatment for conditions such as decompression sickness and carbon monoxide poisoning. Oxygen recently has been found to be an important component in skin rejuvenation, treatment of photoaging skin, and improvement in skin complexions. The interest in the use of HBOT for this purpose is continually growing and becoming more widespread. In addition to aging and genetic makeup, chronic UV radiation due to everyday exposure, especially UV-B, can greatly increase the rate of wrinkle formation through increasing skin angiogenesis and degradation of extracellular matrix molecules. The use of HBOT and hyperoxia conditions has been found to attenuate the formation of wrinkles from UV irradiation. It accomplishes the task by possibly inhibiting various processes and pathways involved such as the HIF1-α, VEGF, neutrophil infiltrations, and MMP-2 & MMP-9, which are directly involved with promoting skin angiogenesis in its active state. There are currently medical aesthetic clinics that are using oxygen therapy under high pressure applied directly to skin to reduce visible wrinkles but this procedure is not widespread yet due to more research that needs to be done on this topic. However, this treatment for wrinkles is definitely growing due to recent studies done showing the effectiveness of oxygen therapy on wrinkles. This review article will explore and summarize researches done on possible mechanisms dealing with the use of oxygen therapy for reduction of UVB-caused wrinkles, its side effects, and its possible future improvement and use in medicine.

## 

Oxygen, at standard temperature and pressure, is a colorless, odorless, tasteless gas. It can exist in its monatomic state, but its preferred state is diatomic, O_2_. It also exists in a triatomic form, or more commonly known as ozone, and is found in the upper limits of the atmosphere [1,2]. Since its discovery, oxygen has been used in treating medical conditions. The first recorded use of hyperbaric air therapy was in 1662 by Nathaniel Henshaw to treat chronic conditions, but it wasn’t until the 1920s when Hyperbaric Oxygen Therapy (HBOT) really received attention and is widely noticed due to previous reports of oxygen toxicity [1]. The first HBOT chamber made in the United States was in 1861 in New York but the most well known chamber was built in 1921 in Kansas [1]. But due to more reports of concentrated oxygen toxicity, HBO therapy was not fully approved and put into practice until the 1937 by Behnke and Shaw for decompression sickness [2]. After the recorded success of HBOT, its use as treatments for numerous medical conditions grew and researches on this subject widen as the treatment has shown itself to be promising. At present, HBOT has grown and became more than just a treatment for decompression sickness. Starting from the late 1950s until now, HBO is used to treat gangrene, stroke, post cardiac arrest patients, and carbon monoxide poisoning [1-3].

## Uses of oxygen for rejuvenation

Joseph Priestley, one of the first discoverers of oxygen, once said, “Who can tell but that, in time, this pure air may become a fashionable article in luxury.” It seems that as the world is developing and Joseph Priestley’s prediction about air is becoming a reality [4]. As the advancement of industrialization, the supply of fresh air is steadily decreasing, making good quality of air more and more of a luxury. Oxygen bar has become available in some big cities such as Los Angeles and Tokyo to provide people with a supply of pure oxygen for a certain fee. These places sell oxygen for recreational uses and different aromas are available for customers to choose from. There are many health benefits claims made by those supporters of oxygen bars. They claimed that the usage could enhance health by strengthening the immune system, reduce stress, increase energy, and reduce headaches and sinus problems. However, specific researches on these oxygen bars claimed benefits have not been done.

Due to the reigning desire in today’s society to maintain youthful appearance, development of minimally invasive dermatological procedures is progressing to rejuvenate aging face. Quite a few of these minimally invasive procedures have been effectively developed such as chemical peels, intradermal fillers, and botulinum toxins, but one not yet fully understood is HBOT [5,6]. HBOT as a therapy for aesthetic means is a relatively new use so there have not been a great number of researches done specifically on usage of oxygen therapy on reduction of wrinkles. However, from the few that has been done, positive outcomes were achieved and the use of oxygen therapy for treatment of wrinkles seems an attractive option [7,8]. Receiving regular treatments of HBOT is thought to increase skin elasticity and stimulate collagen production, leading to reduction of wrinkles and fine lines and improvement in skin texture [9]. Many dermatology clinics and even spas have utilized machines that deliver concentrated oxygen to the patient or client to treat age-related skin problems. Oxygen is used in skin care because it is thought that delivery of natural oxygen increases cell metabolism. The use of oxygen therapy as a process of skin rejuvenation and reduction of loss of elasticity leading to formation of lines and wrinkles are becoming increasingly widespread in skin care clinics because of increasing successful results of their usage due to developing technologies. However, scientific evidences for those claims are waiting to be provided.

## Causes of wrinkle formation

Health of skin is related to whole body health because the skin not only acts as a physical barrier against infections from foreign materials, but also controls the immune system, and produces hormones and neurotransmitters [10]. Wrinkles and aesthetic skin problems, like blemishes and acne scars, are caused by many factors such as aging, exposure to the environment, especially an overexposure to the sun, smoking, gender, and poor nutrition. Wrinkles caused through aging are an intrinsic factor-caused aging, or genetically programmed aging, that happens over time. This genetically programmed aging mainly causes a decrease production of fibroblast, collagen, and elastin, which results in skin wrinkling and elasticity loss [11]. Smoking causes skin aging and wrinkles because tobacco inhibits production of collagen and increase MMP and elastosis production, which degrades matrix proteins important for skin elasticity [12]. Gender wise, skin of women seems to receive more wrinkles than men due to perhaps the estrogen level in women. Estrogen has been found to increase collagen production and skin thickness so as women age with decrease estrogen production, wrinkles formation are more prominent in women than men [13]. As for dietary intake, increasing vitamin C and linoleic acid consumption is associated with slower aging skin, while increasing fat and carbohydrates consumption causes faster skin aging [14].

**Figure 1:**
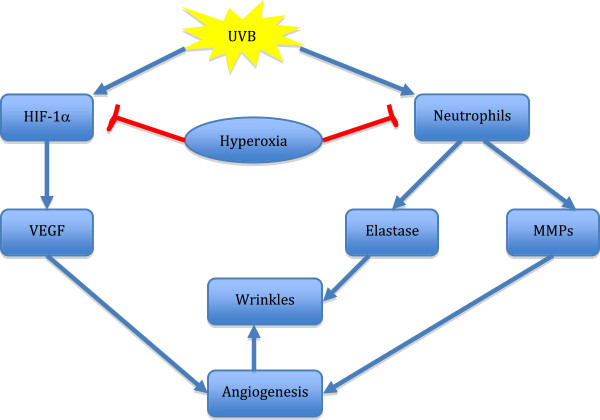
**Overview of possible mechanism used by HBOT to attenuate wrinkle formation from exposure to UVB radiation.** UVB irradiation is shown to upregulate pathways that cause wrinkles such as the HIF-1α, neutrophils, and angiogenesis pathways. Hyperoxic condition is shown to inhibit those pathways activated by UVB, which results in decreased wrinkles formation.

UV radiation causes wrinkles and skin damage, which are symptoms of cutaneous aging or photoaging [15]. Photoaging is characterized by epidermal hyperplasia or atrophy, thickening of basement membrane and stratum corneum, loss of dermal papillae, unusual keratinocytes and melanocytes, degradation of extracellular matrix molecules such as damage to collagen fibers, excessive deposition of abnormal elastic fibers, and increase of glycosaminoglycans. Photoaging is also characterized by dryness, rough texture, abnormal pigmentation, thickening of epidermis, deep creases, and visible wrinkles [16]. UV-B induces matrix metalloproteinases (MMPs), which degrades basement membrane and rearranges the extracellular matrix (ECM), and Type I Collagenase, which digest Type I collagen that is important for supporting the skin, are also causes of wrinkle formation [17]. In addition, it has been found that UV radiation can cause cutaneous angiogenesis, the formation of new blood vessels from pre-existing vessels, that can lead to wrinkles formation by inducing the hypoxia inducible factor (HIF-1) and up-regulation of vascular endothelial growth factor (VEGF) [18,19] (Figure 1).

Angiogenesis has been found to be correlated to wrinkling of the skin and can be caused by not only through UV-B radiation but also through hypoxic conditions. There has been researches done and evidences found of hypoxic conditions leading to wrinkling through angiogenesis by affecting and increasing HIF, which regulates the vascular networks [15]. VEGF is a major angiogenesis factor and a target gene of HIF protein [20,21]. In studies done, the level of VEGF has been shown to be up regulated in areas with necrosis and areas under hypoxic conditions [22-24].

## Mechanisms for HBOT skin rejuvenation

### HIF-1α

As mentioned above, UV-B radiation can cause angiogenesis through inducing the HIF-1 protein, leading to wrinkling of skin. There are two subunits to the HIF-1 protein, α and β. Of these two, the subunit directly involved with hypoxic condition responses is the HIF-1α. The mRNA of HIF-1α is normally made in cells. In some cells, the mRNA level of HIF-1α is increased during hypoxia leading to an increase in transcription of many genes including VEGF. But in most cells under hypoxic conditions, the mRNA level remains the same but the level of HIF-1α protein increases, suggesting that during normal oxygen conditions, the HIF-1α protein usually undergoes proteasomal degradation. The lower oxygen tension stabilizes the HIF-1α subunit and promotes angiogenesis to compensate for the hypoxic condition [21,25].

Since HIF-1α subunits are degraded under normal oxygen levels, this suggests that increasing oxygen tension in the epidermal cells through use of oxygen therapy could increase proteasomal degradation of HIF-1α subunit, which will decrease angiogenesis and slow down wrinkling of skin. The results obtained from studies done by Kawada et al. (26) showed that mice that went through UV-B radiation but receive HBOT did not have a significant increase in HIF-1α protein level like the mice that received only the UV-B irradiation. This result supports that higher oxygen level increases HIF-1α protein degradation and suggests that increase oxygen tension can attenuate formation of wrinkles due to decrease in angiogenesis. Even though the level of VEGF which is a downstream of the HIF-1α protein was found to be increased in both UVB and UVB + HBO group, there was a lower tendency to increase in the UVB + HBO group. Therefore, it is possible that hyperoxia attenuates wrinkle formation through suppressing the HIF-1α angiogenesis-signaling pathway [26].

### MMP-2 & MMP-9

MMP stands for matrix metalloproteinases and these groups of proteins are zinc-dependent proteins involved in remodeling of extracellular matrix and have important roles in angiogenesis, morphogenesis, and metastasis [27]. The protein is made up of several domains, mainly propeptide, catalytic, and hemopexin domains. MMPs are also involved in degradation of collagen, proteoglycans, and many glycoprotein [28]. MMPs are secreted as inactive zymogens (pro-MMP) and have to be activated for full functional capacity. Growth factors and cytokines are molecules that regulate the stimulation or inhibition of pro-MMP synthesis, usually at the transcriptional level. The MMPs involved with photoaging that have shown to increase in level during experiments with human fibroblasts after UV-irradiation are MMP-1, 2, 3, and 9 [17,29]. In other studies done on MMPs in epidermis of hairless mouse skin, after long period of UV-irradiation the levels of MMP-1 and MMP-3 did not have a significant increase. However, the levels of active MMP-2 and MMP-9, in addition to increase pro-MMP-2 and pro-MMP-9 levels, were found to be significantly higher in the UVB-irradiated wrinkled mice skin compared to the unexposed normal mice skin [30]. MMP-2 and MMP-9, also known as gelatinase A and gelatinase B respectively, functions mainly to digest type IV and VII collagens, which are major components of the basement membrane. Also, when a potent synthetic inhibitor of MMP-2 and MMP-9, CGS27023A, was applied topically over a period of time, significant inhibition of reduction of collagen by UVB radiation was observed, and there was no significant difference between collagen levels in non-irradiated mice and CGS27023A-treated mice, which implies that MMP-2 and MMP-9 are have major roles in inducing skin wrinkles after UVB exposure [30].

Even though MMP-2 and MMP-9 have been found to play important roles in inducing skin-wrinkles and angiogenesis, the results obtained in a study from putting UVB-irradiated hairless mice through hyperoxic (HO) conditions did not show significant reduction in the levels of the MMPs [26,31]. The MMP-9 level was found to be the same for UVB and UVB + HO group and the MMP-2 level was found to be slightly decreased in both UVB and UVB + HO group. Because the level of MMP-2 in both the UVB and UVB + HO group were found to be reduced, whether hyperoxic conditions affect MMP-2 levels remains to be elucidated [26]. Perhaps different concentrations of oxygen or different lengths of HBOT exposures need to be adjusted to obtain a more affective result. Another study done on retina of mice showed that hypoxic conditions leads to increase MMP-2 level and angiogenesis through p53-affected CTGF/CCN2 gene of the cysteine-rich protein 61/connective tissue growth factor/novel over-expressed (CCN), which are subsets of extracellular matrix proteins. Suppression of CTGF gene decreased MMP-2 levels and since CTGF gene is induced in hypoxic conditions, hyperoxic conditions could potentially decrease MMP-2 levels and wrinkle formation through inhibition of the CTGF gene [32]. Further research could still be done on this subject to find out if and how high oxygen concentration affects MMPs on a molecular level, which could lead to explaining in more details HBO’s role in wrinkles reduction.

### Inflammatory cells infiltration

Inflammatory cells, especially neutrophils, have the capability to be destructive and can cause damage to the extracellular matrix [33]. Health conditions such as emphysema, adult respiratory distress syndrome, adult periodontitis, rheumatoid arthritis, ulcerative colitis, and blistering skin disorders are mediated by tissue-destructive actions of neutrophils [34-36]. Neutrophils are the most abundant white blood cell and after a certain level of tissue damage experienced, they will quickly leave the blood stream and move towards the site of damage. Exposure to certain degree of sunlight, and an erythemogenic, or erythema-causing, dose of UVB radiation can lead to influx of neutrophils, which cause solar elastosis or break down and loss of elastic tissue [37,38]. The destructive abilities of neutrophils come from the fact that it is filled with potent proteolytic enzymes capable of degrading collagen and elastic fibers, thus causing damage to the extracellular matrix. Neutrophils are able to release a group of serine proteases, mainly the neutrophil elastase, which is a potent proteolytic enzyme [39]. Not much attention has been given to neutrophils regarding its role in causing wrinkles because of more studies explored the hypothesis of MMPs and HIF1 as main causes and also because rate of neutrophil infiltration can only be examined in skin recently exposed to UV radiation [30,40].

In a study done by Rijken et al., they found evidences that infiltrating neutrophils may be the key players in the release of proteolytic enzymes such as MMPs and neutrophil elastase that causes cutaneous damage and photoaging instead of fibroblasts and keratinocytes being the main molecules releasing the enzymes [41]. It has been shown that neutrophils are able to express a few MMPs such as MMP-8, MMP-9, and MMP-12, and in addition the MMP-1 seems co-localized with neutrophil elastase after sun exposure [42,43]. These evidences show that it is likely that neutrophils are the cause of extracellular matrix damage that can lead to elastosis and formation of wrinkles.

UVB-irradiation can lead to angiogenesis in the skin and these additional blood vessels might possibly be the main cause of the increase in inflammatory cells infiltration, which leads to formation of wrinkles. Using HBOT to treat skin wrinkles may be effective because it might be able to decrease the amount of inflammatory cells infiltration and neutrophils releasing the MMPs. Direct evidence was found and shown that hyperoxic conditions are able to decrease blood flow in active muscle cells and also slow down active angiogenesis in the skin [26,44]. Hyperoxia can reduce skin angiogenesis through possibly increasing degradation of the HIF1α protein and with that angiogenesis pathway being inhibited, new blood vessels are not being formed and leads to reduction of infiltrating neutrophils, which results in attenuating the release of MMPs in the skin. However, recent studies have suggested that hyperoxic conditions couldn’t have only affected degradation of HIF1α and cause reduction of wrinkle formation. Evidence was found showing inhibition of HIF1α protein alone was insufficient to restrain wrinkles formation by higher dose of UV radiation that led up to the regulation of the activities of MMPs [45]. With conflicting evidences, more research needs to be done to find out the specific conditions and mechanism of how hyperoxic condition attenuates skin wrinkle formation.

### Thrombospondin-1

Thrombospondin-1 (TSP-1) is a matricellular protein that can inhibit proliferation and migration of endothelial cells, but more importantly in this case, can effectively diminish angiogenesis [18]. Its mRNA is produced by the basal epidermal keratinocyte in human skin and the TSP-1 protein is deposited in the basement membrane area [46]. According to studies done by Kiichiro et al., the epidermal over-expression of TSP-1 inhibits dermal photo-damage and also elastic fiber and collagen disorganization, which lead to prevention of formation of skin wrinkles. They also showed that TSP-1 has a potent ability to inhibit angiogenesis caused by UV-B irradiation by decreasing endothelial cell proliferation and increasing its apoptosis rate [18]. Mice that received chronic UVB-induced skin damage and skin-specific over-expression of TSP-1, compared with mice that only receive chronic UVB-induced skin damage, has greater reduction in skin wrinkling rate associated with the protein’s effective inhibition of angiogenesis.

Recent evidence suggests that the ability of TSP-1 to decrease skin wrinkle not only came from its ability to inhibit angiogenesis, but also its ability to inhibit activation of MMP-2 and MMP-9 by inhibiting conversions of MMPs zymogens to its active form. In addition to degrading basement membrane and rearranging the extracellular matrix, activation of MMP-2 is associated with increased blood vessel growth, while inhibition of this protein leads to decreased angiogenesis level. TSP-1 interacts with MMP-2 by binding to it, which leads to the inhibition of the activity of MMP-2 [47]. It is also thought that MMP-9 has the ability to accelerate wrinkle formation so its interaction with TSP-1 is tested. Results showed that TSP-1 does interact with and inhibits the activity of MMP-9 by binding to it in a similar way that TSP-1 binds to MMP-2 because MMP-2 and MMP-9 has similar structural domains. This property of TSP-1 to inhibit MMPs contributes to its anti-angiogenic effects and also to its ability to reduce UVB-induced skin damage and wrinkles [18,47]. Although TSP-1 has inhibitory activities that can inhibit angiogenesis and slow down wrinkle formation, and hyperoxia conditions and treatments has inhibitory activity on angiogenesis and MMPs level, a direct link between TSP-1 activity and hyperoxic condition has not been made. Apparently more research needs to be done to investigate whether high oxygen tension can increase the level of TSP-1, leading to attenuation of skin wrinkles.

## Conclusion

The use of HBOT in medicine has come a long way since its first main use to treat decompression sickness. In order for the HBOT to be used to its full potential in skin care, the exact mechanisms of how high oxygen concentration reduce formation of wrinkles and photoaging needs to be investigated. The direct mechanism of how hyperoxic conditions can attenuate formation of wrinkles has not yet been established due to conflicting evidences and a need for further research on the subject. The level of HIF-1α protein has shown to be reduced under hyperoxic conditions, which suggests that it is degraded under high oxygen concentration and this can inhibit the expression of VEGF and skin angiogenesis (Figure 1). However, other evidences were found showing that the suppression of HIF1α-angiogenesis pathway under hyperoxic conditions alone is not sufficient to attenuate production of MMPs, angiogenesis, and skin wrinkle formation. Though studies have found HBOT to slow down angiogenesis, others have found HBOT to be capable of promoting angiogenesis in ulcers and wounds [7,48]. More supporting evidence and research is also needed for exactly how high oxygen concentration affects TSP-1 activity and the level of MMP-2 and MMP-9. This is because a direct link between TSP-1 and hyperoxia has not been found and it is not clear yet whether the MMP levels decrease in hyperoxic conditions. A wider variety of different testing conditions need to be enforced to figure out the exact mechanisms. Even with these missing links, the use of high oxygen concentration to reduce the visibility of wrinkles have shown to be promising and effective to a certain degree since this treatment is currently being used and is becoming more widespread in spas and dermatology clinics worldwide.

## Competing interests

The authors declare that they have no competing interests.

## Authors’ contributions

BA did the draft of the manuscript, and JHZ did the revision. Both authors read and approved the final manuscript.
